# Social Media use within medical education: A systematic review to develop a pilot questionnaire on how social media can be best used at BSMS

**DOI:** 10.15694/mep.2017.000083

**Published:** 2017-05-11

**Authors:** William Whyte, Catherine Hennessy

**Affiliations:** 1 Brighton and Sussex Medical School

**Keywords:** Social Media, Medical Education, Facebook, Twitter, Systematic Review

## Abstract

This article was migrated. The article was marked as recommended.

**Background:** Since the early 2000s social media has become a major part of our daily lives, and over the past decade it has found its way into the medical profession. Despite its ubiquity, only 5 systematic reviews exist on the subject of social medial use within medical education. The reviews conclude that there are positive correlations linked to social media use however the studies are restricted by the same limitations: a lack of quantitative data and the fact that social media research fast becomes outdated. This review will therefore examine the latest studies in order to identify which questions remain to be answered and what areas need further development in order for social media to become a credible resource within medical education. The information gained from this process will be amalgamated to create a valid questionnaire which will produce quantitative data.

**Methods:** A systematic review of Pubmed, Cochrane, PsychINFO, ERIC & Scopus was conducted following the Preferred Reporting Items for Systematic Reviews and Meta-Analyses (PRISMA) guidelines. The search was from 1st January 2014 to the 12th January 2017 and included keywords linked with social media and medical education. 27 papers were identified: 12 qualitative and 15 quantitative. From this data a questionnaire was drafted and put to a focus group in order for it to be validated.

**Results:** Six major themes were identified and analysed: community & interactivity, communication & feedback, learning theories, social media vs traditional didactic lectures, role of faculty and professionalism. Quantitative data was limited but highlighted the efficiency of social media use especially when Facebook and Twitter were used. After the analysis a validated questionnaire was produced.

**Conclusion:** Social media can be a useful tool within the medical curriculum if implemented correctly. The final questionnaire can be used to generate quantitative data on the following questions: which platforms are most effective and for what purposes? How beneficial is social media to teaching? and What do students understand the benefits/disadvantages of academic social media platforms to be?

## Introduction

### Social Media and its Platforms

Social media is defined as all “websites and applications that enable users to create and share content, to interact with other users or to find people with similar interests to one’s own” ( Waite and Dictionaries, 2015). The term encompasses multiple platforms ranging from blogs/micro blogs (Twitter ^®^) through to wikis, YouTube ^®^ and social network sites such as Facebook ^®^.

Blogs are the equivalent of online diaries where the author posts in chronological fashion. People who visit these blogs are in turn allowed to comment and reply to the posts (Hollinderbäumer *et al*., 2013). Twitter is considered to be a microblog as each post is limited to 140 characters (Twitter ^®^, San Francisco, CA). According to Cheston *et al.* (2013) blogs are the most widely used form of social media within medical education. These are followed closely by wikis. Wikis are similar to blogs with the exception that all users are allowed to edit the page. The most famous example is Wikipedia ^®^ with over 5.3 million published articles and averaging 800 new articles a day ( Wikipedia, 2017).

Social network sites allow users to create personal profiles online, where they can share information, music, videos, thoughts and opinions. They can be private if the user choses to apply restrictions. This is achieved by choosing specific privacy settings. Alternatively, a public page can be created for all to see (Hollinderbäumer *et al*., 2013). Facebook is the most widely used social network site (Facebook ^®^, Palo Alto, CA). The last major platform of note is YouTube ^®^, which allows users to create and upload videos to the site (YouTube ^®^, LLC, San Bruno, CA). There is then a comments section for people to discuss and share ideas.

### Social Media in Medical Education

Despite the creation of social media’s flagship website ‘Facebook’ in 2004 it has taken over a decade for it to find its way into medical education ( Pander *et al.,* 2014). Currently, according to the literary databases Pubmed, Scopus and Cochrane, there exist only five systematic reviews that assess the role of social media within medical education. Three of them were published in 2013 (Cartledge *et al.,* Cheston *et al.* and Hollinderbäumer *et al.*) whilst the other two were published in 2014 (Pander *et al.*) and 2015 (Roy *et al.*) respectively. The fact that five different systematic reviews were all published within two years of each other is indicative of the current topicality of this theme.

Since the last published systematic review ( Roy *et al.,* 2015) Facebook use has grown by 38%, and currently has over 1.86 billion users worldwide ( Noyes, 2017; Statista, 2016). With a yearly user increase of 17% Facebook, and social media, shows little sign of slowing down ( Facebook, 2017). In conjunction with the exponential growth of social media medical schools are now observing an increase of applicants from the ‘Net generation’ ( Kennedy *et al.,* 2008). These are individuals that have been exposed to digital technologies from a young age and, for the majority, use social media on a daily basis or even as their primary source of information ( Bennett *et al*., 2008; Pander *et al.,* 2014). Whilst most current research is posing the question, “should we incorporate social media into medical education?” it may be more pertinent to ask, “how best can social media be incorporated into medical education?”

### Positive Impact of Social Media

The five systematic reviews address a number of themes from nearly a decade of studies and find that social media use is beneficial when integrated into the medical curriculum. Social media platforms allow for faster feedback between students and faculty members in and outside of the classroom ( Cheston *et al.*, 2013; Hollinderbäumer *et al.,* 2013). This increases the speed of access to information therefore enhancing learning efficiency. The speed and ease of communication was also associated with an increase in student satisfaction ( Pander *et al.,* 2014). This is not only an advantage at the place of study, as the use of social media allows students to transcend geographical barriers, with Cheston *et al.* (2013) finding that students were tweeting academics from other continents and getting replies almost instantaneously.

Throughout the studies, students from various universities highlighted that using social media was a more active process than traditional didactic lectures. They felt more confident in terms of knowledge and more able to readily discuss topics and share their thoughts (Hollinderbäumer *et al.,* 2013). This increased learner engagement and stimulated interactivity between the students, which in turn generated more content and ultimately improved grades ( Cheston *et al.*, 2013). Although the studies addressed in Cheston *et al.*’s review (2013) did not score highly on the Medical Education Research Study Quality Instrument (MERSQI), a tool designed to evaluate quantitative educational studies, these results are still encouraging and merit further research into the use of social media. One of the more rigorous studies found that e-learning was as effective as traditional learning techniques and that social media would build on the positive foundations of e-learning ( Cheston *et al.*, 2013). This is, in part, due to the versatility and customisable nature of social media which can be tailored to the learner’s needs ( Dabbagh and Kitsantas, 2012).

Johnson *et al.* (2011) explain how social media has helped create Personal Learning Environments (PLEs). These are student-designed learning approaches that incorporate various tools (videos, apps, games, pictures..) selected by a student to match their personal learning style and pace. The aim is for students to have an increasing amount of control over how they learn. For example a visual learner would gain more from watching a video on YouTube than listening in a lecture. DiLullo *et al.*, (2011) found that students performed better when they were in charge of their learning. However, PLEs still remain mostly theoretical as they are not widely implemented ( Johnson *et al.,* 2011).

Finally, the systematic reviews pointed out that teaching students how to use social media was good preparation for their professional life. As the world becomes more interconnected, global social media usage is a skill that future doctors will need to master (Hollinderbäumer *et al.*, 2013). The five systematic reviews focused on undergraduate studies but a number of important papers looking at Twitter usage between physicians exist ( Rouprêt and Misraï, 2015; Widmer *et al.*, 2016). Social media is slowly becoming a mainstay of the medical profession. Therefore it is thought that students should be taught how to use it professionally in order to potentiate the benefits whilst simultaneously limiting any complications or unfavourable effects ( Kind *et al.,* 2014).

### Negative Impact of Social Media

Patients are now also using social media to speak to members of the healthcare profession and are more informed than ever. Hollinderbäumer *et al.*’s study (2013) shows the benefits of this by highlighting the knowledge and understanding that students gained from reading about patient’s experiences. There are however many concerns about privacy and professionalism ( Cheston *et al.*, 2013; Hollinderbäumer *et al.*, 2013; Pander *et al.,* 2014). Pander *et al.* (2014) found that 0.2%-16% of students had behaved in an unprofessional manner. Despite the heterogeneity of the results this highlights a widespread issue. The behaviour was linked to Facebook and included various inappropriate statuses, uploading of unprofessional profile pictures as well as confidential information. Students were also members of groups that had criminal connotations. These ideas are briefly echoed in Cheston *et al.*’s study (2013). Patients are known to search for their doctors online. It is therefore important for students to act in a professional manner at all times and maintain their privacy (GMC, 2013; Hollinderbäumer *et al.*, 2013).

Roy *et al.*’s review (2015) found that the negative impact of social media on medical professionalism was the greatest hurdle. These views were felt throughout the profession and meant that many lecturers were reluctant to adopt social media into the undergraduate curriculum. However, Roy *et al.* also found that although concerns over professionalism existed, there was not actually any concrete evidence of unprofessional behaviour when social media was implemented correctly. Cartledge *et al.* (2013) came to the same conclusion and even contacted the authors of the papers included in their study, of whom none could report any actual event of unprofessionalism. There may be however a certain amount of publication bias with editors only publishing articles with positive outcomes ( Cartledge *et al.*, 2013). Further quantitative studies are therefore needed to confirm or dispel the negative connotations linked to the use of social media within medical education. However, even if this is proved to be a drawback of using social media, it is better for students to have the opportunity to hone their professional judgement at medical school. Unprofessional behaviour at such an early stage of their medical career will be less consequential compared to when they are practicing doctors.

Other issues that arise with social media usage are the technical challenges. Firstly there is a discrepancy between the students themselves, with 91% of students aged 18-25 using Facebook, 78% of students aged 26-35 using it and only 6% of over 50s having a profile ( Pander *et al.,* 2014). This suggests that older students might not find social media as useful as their younger peers ( Cheston *et al*., 2013). It is likely that a discrepancy also exists between the level of expertise of the faculty and the students as the staff have not grown up with social media at their fingertips ( Pander *et al.,* 2014). Faculty members are well placed to introduce students to using the various forms of social media at their disposal whilst maintaining a certain degree of professionalism. Many lecturers however do not themselves know how to use social media therefore forgoing the benefits that it could bring to their teaching. In addition, students do not want faculty members involved with their social media profiles ( Pander *et al.,* 2014). This makes it difficult for the lectures to fulfill their potential as teachers and as digital-professional role models.

### Future Development

Cheston *et al*., (2013) write that technologies often evolve faster than the evidence demonstrating their effectiveness. Social media use within medical education is no exception. Whilst the opportunities and benefits of adopting social media into medical education seem to outweigh the cons, the majority of the evidence is descriptive ( Cartledge *et al*., 2013; Pander *et al.,* 2014; Roy *et al*., 2015). There is therefore a need for more rigorous quantitative studies to evaluate its true potential and place within the educational program.

The systematic reviews are also limited in part because the results of this topic are time dependent. The rate of use of social media within medical education is growing rapidly with studies being published on a regularly basis ( Cartledge *et al*., 2013; Pander *et al.,* 2014; Roy *et al*., 2015). This means that once the systematic reviews are available they soon become outdated and fail to address the most current evidence. It is therefore important for regular systematic reviews to be conducted.

### Hypothesis

This review will therefore look at the studies that have been published since the last systematic review was written, of which there are a number of quantitative studies. It is expected that this will add much needed evidence to whether social media should be included in the medical curriculum or not whilst also developing some of the themes that arose in the past five reviews. This review aims to create a questionnaire that will allow the exploration of student opinions and experiences of using academic social media platforms so that information can be gained on how best to use social media within the undergraduate medical curriculum. Ideally, this questionnaire will be dispensed to medical student cohorts at other universities with the aim of creating a larger, more diverse collection of quantitative data.

The research questions this questionnaire seeks to answer are:

• What role can social media play in the medical curriculum; which platforms are most effective and for what purposes?
• How beneficial is social media to teaching; is it equal to or more effective than traditional educational sources?
• What do students understand the benefits/disadvantages of academic social media platforms to be?

## Methods

A systematic review was conducted following the Best Evidence Medical and Health Professional Education (BEME) protocol ( Flannery, 2015) and Preferred Reporting Items for Systematic Reviews and Meta-Analyses (PRISMA) guidelines ( Moher *et al*., 2009).

### Search Strategy

Using the methods described by Cook and West (2012), two medical education researchers, the following databases were used: Medline, Cochrane, PsychINFO, ERIC (Educational Resources Information Centre [for education studies]) and Scopus. The search was conducted from 1 ^st^ January 2014 to 12 ^th^ January 2017. 2014 was chosen as a cut off date because it represented the year that the latest systematic review was accepted by its publisher (Academic Psychiatry) ( Roy *et al.*, 2015). Papers from this point therefore would not have yet been examined. Any paper that had already been reviewed was excluded from this study after cross-referencing the bibliographies from the five existing systematic reviews.

The search terms were *social media, social networks, Web 2.0* as well as the two largest social network sites *Facebook* and *Twitter.* These were in combination with *medical education* and *medical student education.* The resulting search on Medline was:

“Facebook”[All Fields] OR “Twitter”[All Fields] OR “Web 2.0” [All Fields] OR “social media”[All Fields] OR “social networks”[All Fields] OR “social networking”[All Fields]) AND (medical education [MeSH Terms] OR “medical student education”[All Fields])

In addition to this, the reference lists of randomly selected articles were hand-searched to identify additional articles; this would continue until no additional articles were identified. The first paper was examined with no further articles meeting the inclusion criteria ( Rodríguez-González *et al.,* 2016). Three more bibliographies were analysed in case the first was an exception but again no pertinent studies were identified ( Brisson *et al.*, 2015; Ekarattanawong *et al.*, 2015; Sood, 2015). The search resulted in a total of 1056 studies from the databases as follows:

• Pubmed (Medline): 294 studies
• Cochrane: 96 studies
• PsychINFO: 9 studies
• ERIC: 99 studies
• Scopus: 566 studies

### Inclusion & Exclusion Criteria

All article types were included in this review including both peer reviewed and non-peer reviewed research. It was felt that many pieces of grey literature were relevant for the purpose of this study and could add to evidence-based decisions as long as their limitations were recognised ( Cook & West, 2012).

Articles were excluded for the following reasons:

1. Postgraduate study - The focus of this review was undergraduate medical studies. This meant that all studies conducted post-medical school were excluded.
2. Non-medical education - All papers that looked at the use of social media in non-medical education were excluded.
3. Date - As mentioned previously 1 ^st^ January 2014 was chosen as a cut off point.

To minimise bias a second author, Catherine Hennessy (CH), checked the terms of exclusion and agreed with the parameters for all included article

### Study Selection

**Figure 1.**
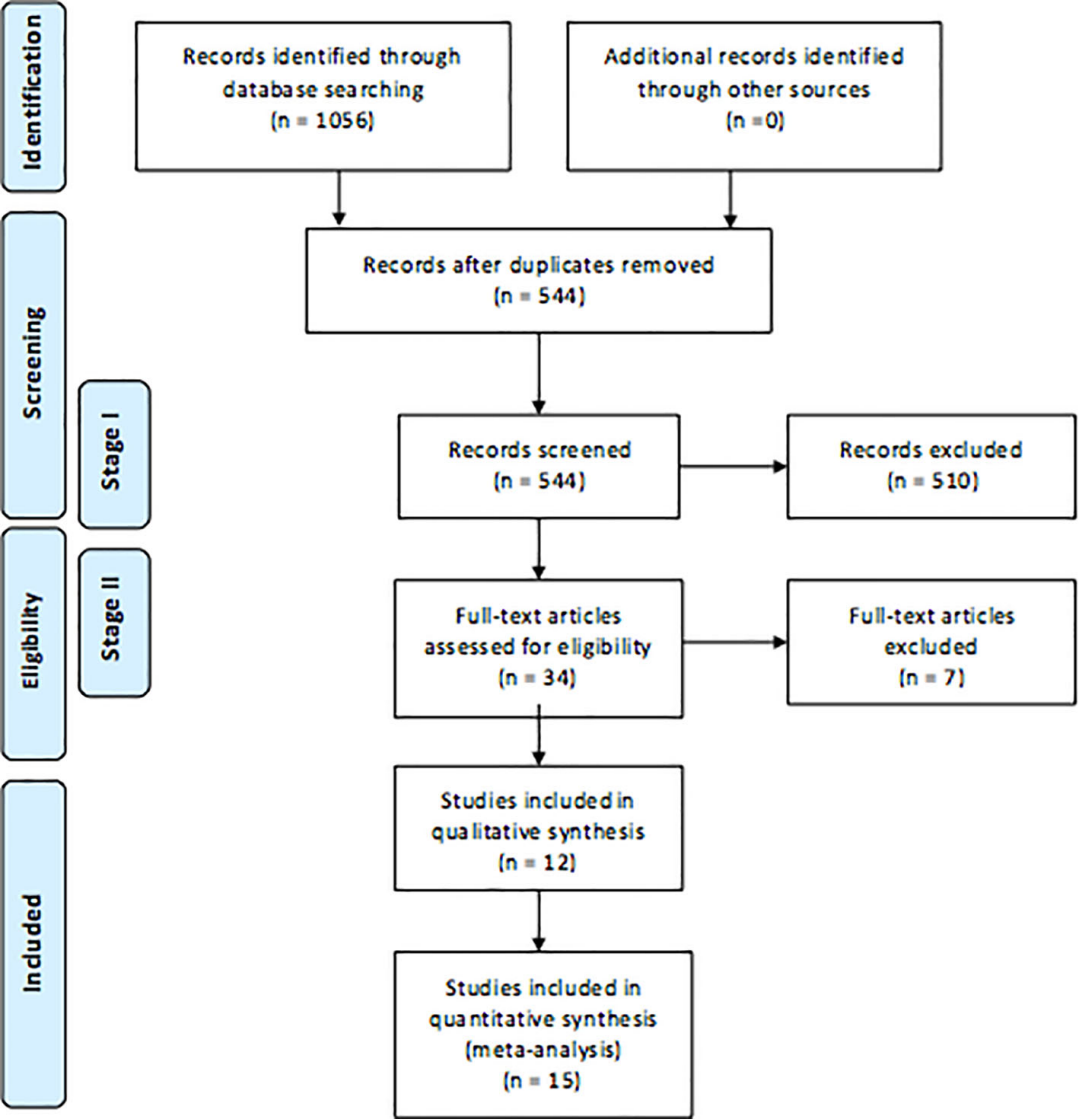
Flow diagram: Study selection and evaluation

The study selection was conducted in two stages. Firstly, articles were excluded after screening the article’s title and abstract. If there was any ambiguity the paper was reviewed in the second stage. During the second stage papers were read in their entirety before being excluded. The resulting process can be seen in the flow diagram ( Figure 1). The template was taken from the PRISMA website with its eligibility having been reviewed multiple times ( Moher *et al*., 2009).

### Data Extraction

Thematic analysis was conducted with ‘Mendeley’ software ( Mendely Ldt, 2016). Important information was highlighted and coded then grouped together in themes. Information was put into tabulated form along with the study’s limitations and conclusions ( Appendix 1, 2 & 3). This task was undertaken by the primary author (WW) and verified by the second author (CH). Both qualitative and quantitative studies were included.

### Questionnaire creation and validation

After reviewing the literature a questionnaire was constructed following Boynton’s (2004) guidelines ( Appendix 4). The aim was to create a questionnaire that could be used universally to generate a large archive of quantitative data. The questionnaire was reviewed and validated in two separate focus groups by a total of eight students. The questionnaire itself was divided into four separate sections, each one tailored to answer specific questions.

Part 1 was created to gage how useful social media was compared to traditional learning materials such as lecture slides, texts books and core reading lists. Part 2 focused on which social media platforms were used, how frequently and whether this was for social, educational or professional purposes. Part 2 also established which features of social media would be most useful if used within medical education. Part 3 addresses a limitation that occurred in a number of the included studies: professionalism. Finally, Part 4 was added as an open question at the end so that students could highlight any areas that might have been overlooked.

## Results

### Qualitative and Quantitative Studies

The initial database search resulted in 1056 papers. After duplicates were removed only 544 remained. The first exclusion phase removed 510 papers and the second phase removed 7. The result was 27 different papers of which 12 were qualitative and 15 were quantitative ( Figure 1).

The remaining papers were analysed and the key information was put into table format. This included study design, data type, study limitations and conclusions ( Appendix 1 & 2). The coded information was grouped into six themes based on the areas of impact that social media had within medical education ( Table 1).

**Table 1.**
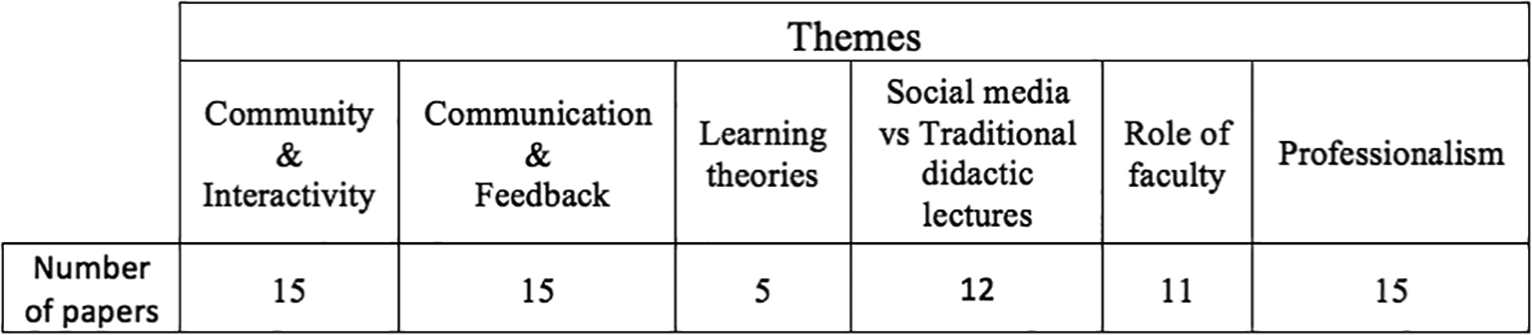
The 6 identified themes with the number of papers they feature in

### Questionnaire

The original questionnaire ( Appendix 4) was created based on the themes identified over the course of this review. After it was designed eight Brighton and Sussex Medical School (BSMS) students completed the questionnaire. Four of them were in their first year of study whilst the other four were in their fifth year. Upon completion feedback was collected and appropriate changes were made to the questionnaire ( Appendix 5).

Part 1 remained largely unchanged apart from ‘offline multimedia’ being replaced by ‘journals’. Originally ‘offline multimedia’ was supposed to represent journals, books and papers that could be accessed offline, at the university library for example. The participants of the focus group said however that this was not clear and that if they were going to access any information other than a textbook it would be an online journal.

In Part 2 a definition of social media was added as there was a discrepancy between what the participants believed social media to be and the actual definition. For example all eight members were unaware that Wikipedia and YouTube were social media platforms and this affected their response to question 1 of Part 2. These two sites were therefore added to the definition. There was also some confusion over the acronym “SMS” as the majority of the participants thought this was referring to text messages also know as Short Message Service (SMS). Social media sites therefore became Social Media Platforms (SMP).

In Part 2, question 3 several comments were made on the need to have a time frame between ‘once a week’ and ‘once a day’. This was also evident with some participants circling the line between the two boxes. ‘Several times a week’ was therefore added to provide the detail needed. In question 4 of Part 2 the participants though that ‘Webinars’ should also be included, as they would find them useful.

In Part 3, two participants felt that value would be added to the study if the questionnaire asked which SMP they had a personal and private account on. An asterisk and question were added to the table. Finally, throughout the questionnaire the term ‘professional’ posed a problem. At undergraduate level it seems that education and profession are synonymous. This was also found to be the case in Usher *et al.*’s study (2014) with only a small proportion of final year students (11%) having a professional LinkedIn account. Due to the ambiguity it caused it was removed from the corresponding tables.

## Discussion

The use of social media within medical education is in its infancy. Previous systematic reviews have found that is it a useful resource and can be beneficial when implemented correctly. Many of the studies however are still only descriptive and there is a need for quantitative date in order to justify social media’s place within the medical curriculum. Through an examination of the most up to date literature this systematic review aimed to evaluate three of the main social media platforms: Facebook, Twitter and YouTube and how they have been integrated into the medical curriculum. Secondly, the six recognised themes ( Table 1) were analysed in order to identify which questions remain to be answered and what areas need to be developed further in order for social media to become a credible resource within medical education. The information gained from this process was amalgamated to create a valid questionnaire intended to produce future quantitative data.

### Platforms used

#### Facebook

Various social media platforms were used across the studies. Facebook was the most popular as it was used in nine studies followed by Twitter (7 studies), YouTube (5 studies), wikis (2 studies) and blogs (1 study). Of the nine Facebook studies four of them were quantitative with a combined cohort of 1556 medical students from over 12 different countries ( Amgad and AlFaar, 2014; Ekarattanawong *et al.*, 2015; Jaffar, 2014; Usher *et al.*, 2014).

A large portion of the reviewed student cohorts use Facebook. This ranged from 78.8% to 93% of students ( Amgad and AlFaar, 2014; Usher et al., 2014). In Jaffar’s study (2014) he shows an increase of usage over the course of one year. In 2012, 86% of students were actively using social media, in 2013 this rose to 92%. Amongst users however there were found to be some discrepancies between age groups. Usher *et al.* (2014) found that whilst 93% of first year students used Facebook, the majority of these were aged 16-25 (97%) with only 74% of students aged 45 or over accessing the site. Facebook’s influence is steadily growing with most students actively using the site. Despite the potential lack of familiarity amongst older students Facebook has the potential to be a useful tool.

Facebook was used in a number of ways. Jaffar (2014) created a ‘Human Anatomy Education Page’ (HAE). The most popular feature of this page was pictures of anatomical structures that were uploaded and that students then had to identify and label. 96% of students used this feature. The second most used item (94%) was multiple-choice questions followed by explanatory comments (88%), videos and video links (87%) and links to other online anatomy resources (82%). Ekarattanawong *et al.* (2015) and Amgad and AlFaar (2014) adopted a different approach. They used Facebook more as a means of communication that allowed them to keep in contact with students with updates on day-to-day requirements. Facebook is therefore a multifunctional tool that would allow educators and students alike to adapt to their own teaching and learning styles.

The results were unanimously positive. Jaffar (2014) found that 84% of medical students “agreed/strongly agreed that Facebook could be a suitable learning environment”. Amgad and AlFaar (2014) reported that 98.1% of students said that they would “recommend the use of social media” and that 96.2% agreed that the use of social media “made the course more intellectually stimulating than if it was based on conventional methods”. In similar fashion Ekarattanawong *et al.*, (2015) found that the majority of students wanted social media to be integrated into their other modules.

More specifically, students found that social media made learning more interesting and challenging, whilst improving their self-confidence and understanding. It also allowed students to communicate with tutors and colleagues more openly and instantaneously ( Jaffar, 2014; Sood, 2015). It can therefore be seen as a supplement to conventional teaching. Despite the mild heterogeneity in terms of Facebook use between age groups it is still widely used. As most students use it for socialising/entertainment this means that its transition into academia should be straightforward ( Sood, 2015). This aligns with Malcolm Knowles’ ‘andragogy’ theory that states that adults learn more efficiently when they integrate familiar tools into their learning ( Barry *et al.*, 2015) suggesting that by introducing social media into the curriculum, learning should become more effective for students.

Whilst the results of these studies are promising, there exist several limitations. The main issues center around the format of the four quantitative cross-sectional studies ( Amgad and AlFaar, 2014; Ekarattanawong *et al.*, 2015; Jaffar, 2014; Usher *et al.*, 2014). Several of them are prone to sample bias. Usher *et al.*’s (2014) cohort was 82% female, Jaffar’s (2014) was made up from a small group of students from the United Arab Emirates and Sood (2015) only looked at the opinion of one educator. This reduces generalizability. However, the same outcomes are being noted across the different studies so by pooling the results together the sample bias is reduced.

As most of these studies are explorative in nature they lack methodical rigour. The qualitative studies are often limited to one or two authors and therefore prone to researcher subjectivity whilst the quantitative studies are prone to response bias ( Amgad and AlFaar, 2014; Gaglani and Haynes, 2014; Guarino *et al.,* 2014; Madanick, 2015 and Usher *et al.*, 2014). This is generally because the test subjects are volunteers and are computer literate. The results of the self-reported questionnaires are therefore more likely to portray social media in a positive light. For more rigorous results, future studies will need a more diversely selected cohort and ideally a control group to compare social media and traditional teaching methods.

#### Twitter

Seven of the included studies looked at Twitter and how it can be used within medical education. When Junco *et al.* (2011) integrated it into the curriculum they found that students were more engaged in the subject and achieved better exam results. This was thought to be because of the improved communication between the students themselves and staff. Webb *et al.* (2015) also found that the students that participated in the weekly Twitter quiz had improved exam results compared to those that did not. To our knowledge this is the only study that compared Twitter users with a control group. This is therefore a more rigorous study and adds weight to the argument in favor of incorporating social media into the medical curriculum.

Hennessy *et al.* (2016) however found a negligible positive correlation between Twitter use and grade results, despite 91% of the cohort using the Twitter hashtag. Yet they found that it increased student engagement during the anatomy course as well as creating a support network that helped reduce anxiety and stress. As it is a microblogging platform, it is well suited to ongoing public dialogue. Hennessy *et al.*’s hashtag created an online, informal community where students could share their thoughts and concerns, which in turn encouraged learning. This was also found to be the case in Madanick (2015) and Chretien *et al.*’s studies (2015) as students used it for networking opportunities, mentorship and learning.

It must be noted that the regular input of teachers was needed to successfully manage the Twitter account as a support tool ( Hennessy *et al.*, 2016). This could be seen as a disadvantage as staff would have to work extra hours. Alternatively, the time invested by the staff in Twitter could be offset against the time it would take to individually respond to student’s emails, as Twitter offers a ‘one-to-many communication channel’ ( McArthur and Bostedo-Conway, 2012). Hennessy *et al.* (2016) also noted that the mean student ratings for the anatomy workshops had significantly increased since the introduction of the anatomy Twitter account and specific neuroanatomical hashtag suggesting a positive correlation between social media use and student satisfaction. However, the mean was also found to have increased over the two years preceding Twitter’s inclusion in the curriculum. It is therefore difficult to note accurately if Twitter led to the significant increase or if it was just a continuation of an existing trend.

Usher *et al.* (2013) compared Twitter and Facebook use and concluded that Twitter use is comparatively low. Only 14% of first year students and 16% of final year students used Twitter. In terms of global use, by the end of 2013 Facebook had 987 million more users than Twitter yet, over the course of the last three years they have however had the same growth of 150% ( Statista, 2016). It therefore remains a substantial element of social media and should not be ignored. It is also widely used by healthcare professionals to track worldwide conversations in order to gain a better understanding and wider perspective on chosen topics ( Rouprêt and Misraï, 2015; Widmer *et al.*, 2016; Wilson *et al.,* 2013). O’Kelly *et al.* (2015) reported that students found the Twitter account ‘@surggrandrounds’ extremely useful. It made the information from their surgical teaching more accessible and the students wanted Twitter to feature in their other modules.

Most studies found that Twitter and social media in general are a welcome addition to traditional lecture based learning. It remains to be seen how useful it can potentially be and how it fares in comparison to traditional teaching methods. Part 1 of the questionnaire was tailored to address this topic ( Appendix 5). Indeed it asks how useful students find various learning materials and quantifies the answer. With the resulting information social media will be comparable to other methods such as textbooks and lecture slides. Webb *et al.* (2015) concluded that Twitter is not a replacement for existing aspects of medical education but that it should be considered as a useful adjunct to the curriculum as students found it added to their education. There were however questions raised over its utility given that each Tweet can only be 140 characters long ( Hennessy *et al.*, 2016). Moreover, there remains some concern that this short style of communication will encourage poor writing habits and grammar among students ( Grosseck and Holotescu, 2008). However, this issue was not reported in any of the included studies and it can be argued that character limit allows the information to be concise and therefore more beneficial.

As with Facebook, there were many limitations surrounding these early studies. Again responder bias and sample bias are the main issues, for example Chretien *et al.*’s study (2015) consisted of Twitter ‘superusers’. This meant that Chretien *et al.* chose students that already had Twitter accounts and more specifically students that used it to access professional content. Although, due to the ‘superuser’s’ expertise, this did show how Twitter could be used to its full potential, it is not representative of what the average medical student would do. These users would also have been biased during their interviews as they inevitably would have responded in Twitter’s favour. Usher *et al.* (2014) and Webb *et al.* (2015) also had cohorts made up of proficient Twitter users which could have skewed results. In addition to this, the majority of the studies failed to include a control group and therefore could not compare social media use with other teaching methods ( Amgad and AlFaar’s, 2014).

#### YouTube

YouTube was also documented as being an important learning resource ( Amgad and Alfaar, 2014; Barry *et al.*, 2016; Madanick, 2015; Rabee *et al.,* 2015). The video format meant that students were able to view and visualise concepts therefore heightening their understanding. This was particularly important for subjects such as anatomy (Barry *et al.*, 2016). In Jaffar’s 2012 study he found that 92% of students “agreed” or “strongly agreed” that the Human Anatomy Education channel on YouTube was important to their understanding of the subject. Barry *et al.*, (2016) found that 78% of participants used YouTube as their primary source of information for anatomy with only 29% looking at recommended textbooks. With such a large proportion of students using YouTube it is important that lecturers adopt this tool.

The rise in popularity of YouTube, and by extension social media, is due to the speed of access of information. The majority of students at undergraduate level are Millennials that operate at ‘twitch speed’ ( Prensky, 2004). They expect responses and feedback instantaneously. This, paired with the three dimensional qualities of YouTube, make it an invaluable tool for learning anatomy (Barry *et al.*, 2016). The major issue with YouTube is that the material the students are viewing has not been validated. Inevitably there will be students learning information that is incorrect or misleading.

This is an important concern throughout the literature and not unique to YouTube ( Guarino *et al.,* 2014; Sherbino and Frank, 2014; Sherbino, 2015). Students must understand that not all resources are equal. Lecturers therefore have a duty to warn students about these dangers. Ideally, staff could produce their own videos or at least evaluate and share the most pertinent online material ( Madanick, 2015 and Rabee, 2015). Chretien *et al.* (2015) suggest that students should be taught how to critically evaluate the information that they are accessing via social media. If not, they risk leading themselves and others astray ( Rodríguez-González *et al.,* 2015).

This review chose to focus on Facebook, Twitter and YouTube as they featured in the majority of the included studies. As mentioned earlier these sites are extremely popular among students socially, but that does not mean that they are the best educational resources. Other social media platforms may be just as beneficial but have been overlooked in research due to the popularity of Facebook and Twitter. Part 2 question 2 of the questionnaire ( Appendix 5) therefore addresses this issue. Six other social media platforms are put forward alongside Facebook, Twitter and YouTube in order to see if they are also used either socially or educationally. The following section of the questionnaire then allows students to expand on how they use these platforms with question 3 gauging the frequency of use.

Various methods have been used across different platforms to try and integrate social media into the medical curriculum. The results and feedback from the students has been positive despite the heterogeneity of the techniques. Part 2 question 4 of the questionnaire ( Appendix 5) was therefore created to generate quantitative data on which features of social media would be most beneficial to students. This information will hopefully give insight into the areas of social media that will be most valuable to students and consequently adopted by educators.

### Community & Interactivity

Social media helps bring groups of people together, both students and faculty, leading to a stronger sense of community, which in turns increases interactivity, productivity and confidence ( Amgad and AlFaar, 2014; Chretien *et al.,* 2015; Duke *et al.*, 2015; Hennessy *et al.*, 2016; Jaffar, 2014 and Sherbino, 2015).

Chretien *et al.* (2015), Hennessy *et al.* (2016) and Sherbino’s (2015) papers all elucidate this sense of community and its importance. Chretien *et al.* and Hennessy *et al*. show how through Twitter medical students are able to bond by supporting one another through the rigours of medical school. Faculty were also found to provide guidance and encouragement through Twitter. Communities are no longer bound by geography as students also followed groups of like-minded people outside of their medical schools ( Hillman and Sherbino, 2015). These virtual communities serve as sources of inspiration. Chretien *et al.* (2015) recorded one student’s wish to pursue a primary care specialty following the information and experiences she’d gained from one online community.

The most striking example of virtual communities is Free Open Access Meducation (FOAM) ( Hillman and Sherbino, 2015; Madanick, 2015; Sherbino and Frank, 2014). FOAM is a collection of resources and tools as well as a community and ethos. Twitter has been instrumental to its success and development. It is a symbol of what social media can achieve within medical education. Its goal is to distribute information and resources around the world with its philosophy derived from the Hippocratic oath: ‘[..] to teach them this art - if they desire it - without fee and covenant’ ( Nickson and Cadogan, 2014).

The nature of social media and therefore FOAM allows for locally produced information to be distributed around the globe. One emergency medicine blog featuring on FOAM had as much as 12 million unique visits a year ( Cadogan *et al.*, 2014). Sherbino (2015) noted that this ‘virtual participation’ helped to enrich the wider discussion of a topic. Free access to such large pools of educational resources can only serve to benefit students and educators alike. Webb *et al.* (2014) showed that it helped improve student grades. It can therefore be argued that instantly accessible learning resources like FOAM should complement the medical curriculum.

Despite this success there is some negativity surrounding FOAM. This is because the information is not peer reviewed like traditional medical journals and could therefore potentially be misleading or false. However, given the size of the FOAM community articles are, in their own way, reviewed. As all publications are free they are open to debate and discussion by the entire medical community. For the most viewed resources the scrutiny to which they are subjected could arguably be more rigorous than the traditional peer-review process. Unfortunately this is not the case for all information and students must be selective in what sources they chose to view ( Parsi and Elster, 2015).

Social media has revolutionised the way we produce and distribute information. It should therefore start to find its place alongside journals and textbooks as an educational tool ( Nickson and Cadogan, 2014). As well as an increase in recourses social media offers greater interactivity, a greater understanding of learning responsibility and a means of continuous feedback about one’s own progress in comparison to peers. Social media serves to inspire and engage students as well as improving understanding and widening their perspectives ( Amgad and AlFaar, 2014; Usher *et al.*, 2014).

### Communication & Feedback

With community comes communication. Social media allows for faster communication and feedback from peers but more importantly from lecturers allowing students to act in a more timely and productive fashion ( Ekarattanawong *et al.*, 2015; Usher *et al.,* 2104).

Ekarattanawong *et al.* (2015) explore this further in their study. They show that social media enhances communication with lecturers. This is because they can speak directly to a staff member whereas before they had to go through a class representative. The speed of response has also increased. It was found that questions answered by means of social media were more valued than an in class answer. Controversially, Ekarattanawong *et al.* (2015) found that communication between students themselves was poor. This could be because the use of a Facebook page is not as suited to open discussions as other forms of social media. The authors suggest that a ‘closed group’ would yield better participation.

The speed of feedback was also noted by Hennessy *et al.* (2016) to be a strength of social media and in particular Twitter. Several students reported that it helped over the revision period as feedback was rapid and concise due to the 140-character limit. In addition the hashtag being used was public and therefore available to all students. This meant students were able to read each others questions and more importantly the feedback they received from staff. This was noted by most students to be a vast improvement to emailing lecturers. However, several students felt that on occasion the 140-characters was not enough for a detailed explanation and stressed that the option to email should still be available. Again, this is a case of how social media can complement existing tools to improve the medical curriculum.

Hennessy *et al.* (2016) also noted that the face-to-face relationship between student and lecturers was improved. Students felt that because they had spoken to staff via Twitter that they were then more approachable in the lab. This shows that a relationship built online can be transferred to the classroom. Conversely some students found that they did not know how to address their lecturers via Twitter. Therefore if social media is chosen to be part of the curriculum then lecturers must first set out guidelines so students know how to utilise this new tool.

### Learning Theories

There are two key learning theories that underpin the use of social media within medical education: Connectivism and Constructivism (Davis *et al.,* 2015; Flynn *et al.*, 2015; Hennessy *et al.*, 2016; Mi and Gould, 2014). However, a large number of educators are unaware of their importance despite their relevance. Connectivism explains how Internet technologies have created new opportunities for people to learn and share information across online peer networks ( Siemens, 2005). Constructivism is an umbrella term that groups together a number of learning theories that have become more prominent since the birth of social media. They are centred on the fact that students subjectively construct knowledge themselves. Flynn *et al.*, (2015) identified the Social Development Theory and Communities of Practice to be the most important Constructivism theories linked to social media whilst Hennessy *et al.*, (2016) highlight the importance of a Zone of Proximal development ( Table 2).

**Table 2.**
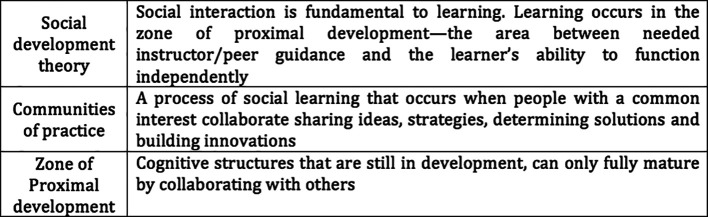
Definitions of Social Development Theory, Communities of Practice and Zone of Proximal development

With the integration of social media into medical education, learning has become a more social process because it is user-generated and collaborative. Students are able to build their own knowledge from people with more expertise than themselves. This traditionally is the lecturer but can also include peers. It is thought that this active process of understanding through interactions with others is vital for the students’ development and learning ( Flynn *et al.*, 2015; Hennessy *et al.*, 2016). This could be the reason that exam results were better in Junco *et al.* (2011) and Webb *et al.*’s (2015) studies. However this remains a hypothesis until it becomes the subject of its own study.

Connectivism mirrors the Constructivist theory for learning but focuses specifically on using social media as an educational tool and in doing so enhances the experience. It is easier for lecturers to connect learners to one another via social media, which allows for an active learning environment. Students are more able to share information, ideas and feedback especially outside of the classroom. Via social media they are also able to contact experts and are therefore not limited to the resources at their own medical school ( Hillman and Sherbino, 2015). Lecturers that use social media should have an understanding of Connectivism and Constructivism. This will help them plan effective learning events and assessment practices which will ultimately enhance the students’ learning ( Flynn *et al.*, 2015).

### Role of Faculty

Social media is a relatively new tool and can seem alien to faculty members. Barry *et al.,* (2016) highlights that 63% of surveyed educators did not want to use Facebook as a teaching tool whilst 85% had had no formal training. This is problematic as lecturers need to be familiar with what the students are doing and in certain cases even teaching them how to best use social media. Several of the studies proposed how lecturers could achieve this as well as analysing their interactions with the students ( Duke *et al*., 2014; Flynn *et al*., 2015; Hennessy *et al*., 2016; Kind *et al.* 2014).

Students are starting to use social media to supplement their learning and some even use it as a primary source of information ( Usher *et al.*, 2014). It is therefore important for faculty members to engage with these tools so that they can ensure that the viewed material is both correct and fit for purpose or even highlight high-quality resources to learners (Barry *et al.*, 2016; Walji and Stanbrook, 2015). Lecturers could go a step further and produce their own material. Raikos and Waidyasekara (2014) reported that a faculty-produced video was found to be extremely useful by 92% of surveyed students. This proves that institute led material has the potential to be a high yielding learning source and that faculty should be encouraged to prepare their own material online.

Another important aspect of lecturers being on social media is that they can share their material with a larger audience. They can then get feedback from students and teachers worldwide and subsequently improve their content ( Madanick, 2015; Walji and Stanbrook, 2015). They can also use the ‘insight’ tools offered by the various platforms that provide analytic data. Madanick (2015) uses YouTube as an example. Lecturers that have posted videos online can find out how many times they have been viewed, if the entirety of the clip has been watched and whether segments have been replayed. With this data they can then adjust their videos accordingly. If sections have been replayed for example this might suggest that the subject matter was more complex in nature and would benefit from a more in depth explanation. This type of feedback is quick and efficient and allows for improved teaching material.

This tool is not unique to YouTube and can also be found on Facebook and Twitter which allows the administrators to track page interactions and popularity ( Ekarattanawong *et al.*, 2015; Gaglani and Haynes, 2014; Hennessy *et al.,* 2016; Jaffar, 2014). These activity measures however are limited to the administrator and their activity. For example Twitter Activity can only track the user’s tweets and not the tweets or comments made by others ( Hennessy *et al.,* 2016).

Hennessy *et al.* (2016) stress the importance of faculty involvement as without their input social media as an educational tool would not be effective. In their study members of staff maintained a Twitter account. For it to be useful they had to view it several times a day in order to answer the students’ questions. As mentioned previously this took less time than answering individual emails as the answers on Twitter were visible to the whole cohort.

For this to be achievable staff members may need to have some form of IT training as they are not as apt at social media use as students ( Duke e *t al.*, 2014). Brisson *et al.*’s (2015) study focuses on the mismatch between lecturer and student. Whilst they believe that it is the lecturer’s role to teach students how best to use social media, they found that the staff members did not actually have the skill set to do so. If social media is misused then it can negatively impact the students learning due to disrupted workflow and the potential to distract ( Flynn *et al.,* 2015; Madanick, 2015). If social media is to be successfully integrated into medical education than this mismatch must be addressed. Faculty must be as at ease with social media as the students ( Jaffar, 2014). These points are raised in Kind *et al.*’s (2014) study which is a compilation of twelve tips that help take educators through the process of using social media within medical education.

Flynn *et al.* (2015) outlined what lecturers can and should be achieving with social media usage. The study concluded that lecturers should have an understanding of Connectivism and Constructivism so that they could provide the most effective education. To do this they should provide scaffolding to learners with greater support at the outset of learning. This can be achieved by sharing links to resources via Twitter or maintaining the module Facebook page. The lecturer should then take a step back to allow the learner to developed their own knowledge and expertise. They could do so by testing the students’ knowledge with online quizzes, multiple choice questions or polls. These are all achievable via Twitter and Facebook.

There exist concerns over mature students and staff’s aptitude at using social media. The questionnaire was tailored to gather information from students but insight could be gained if lecturers also completed it. The demographic section paired with Part 2 question 1 would indicate how apt people are at using social media according to their age. Lecturers could also complete questions 2 and 3 of Part 2 which would indicate which platforms they use, if any, and how often they use them. Question 4 would help gather information on what the educators think would work best in terms of teaching and this could then be compared to the responses given by the students.

### Professionalism

There are many positives surrounding the use of social media within medical education however there is a lot of concern over professionalism ( Chretien *et al.,* 2015; Gooi *et al.,* 2014; Hennessy *et al.,* 2016; Jaffar, 2014; Parsi and Elster, 2015; Raikos and Waidyasekara, 2014; Shamdas *et al.,* 2014; Walji and Stanbrook, 2015). This includes conflicts of interest, privacy and confidentiality violations and inappropriate relationships with patients. The online environment is a new domain that is not yet well structured or regulated. The potential for social media use to backfire is ever present. As much as 60% of medical schools in the USA have reported incidents of unprofessional behaviour and more than half of students have described unprofessional behaviour by their colleagues on Facebook ( Brisson *et al.* 2015). There exist documented cases of sanctions and expulsions from medical school ( Chretien *et al.,* 2009; Greysen *et al.,* 2012).

In order for the patient-health care professional relationship to succeed privacy and confidentiality must be maintained, since preserving a patient’s trust is fundamental to their care ( Parsi and Elster, 2015). Privacy and confidentiality differ slightly in their terminology. Privacy is defined by the individual; they can divulge or withhold whatever information they choose. It is patient controlled. Confidentiality on the other hand is controlled by the professional. Information has been volunteered by the patient and it is the healthcare professional’s role to protect it. It is important to differentiate these two words as both are affected by social media use but in different ways. Medical students may be lax with their own privacy, at their own cost, but cannot afford to compromise a patient’s confidentiality ( Sood, 2015).

There exist national guidelines as well as university guidelines on how to behave online. These include points on safeguarding patient privacy, avoiding controversial material and “pausing before posting” ( BMA, 2011; Davis, 2015; Hillman and Sherbino, 2015; Parsi and Elster, 2015; Usher *et al.,* 2014). This is in place so the students will reflect on the fact that they are posting in a public domain. However, students are not often aware of these guidelines or in some cases disagree with them. In Chretien *et al.*’s study (2015) the Twitter ‘superusers’ disregarded the idea of having separate professional and personal accounts. They felt that the personal aspect added authenticity to their account. This was especially important for them when conversing with patients. This however seems to be an irregularity.

Most health care professionals want to keep their personal and private lives detached and this is mirrored in the guidelines ( O’Kelly *et al.*, 2015; Hillman and Sherbino, 2015; Parsi and Elster, 2015). Unfortunately with most social media platforms it is often difficult to have two separate accounts. This shows a discrepancy between the guidelines and what is actually achievable. Due to the fact that social media is a relatively new area, there seems to be a lack of understanding from the governing bodies. On top of this, due to the ever-changing nature of social media, any relevant guidelines fast become outdated.

Chretien *et al.* (2015) conclude that the participants used Twitter with thoughtfulness and purpose. Their behavior was exemplary and any concerns about unprofessional behaviour were unfounded in this cohort. However this study did include Twitter ‘superusers’ so their behavior may not be representative of the average medical student as the ‘superusers’ were found to be more aware and conscientious of their actions.

For the general student then it is important for educators to integrate ethics and professionalism into their teaching as two thirds of students had noticed unprofessional material on their peers’ social media profiles ( Brisson *et al.*, 2015). These were often not acted upon despite conflicting with the guidelines. This is thought to be because the current net generation sees their social media profiles as an extension of themselves and confronting someone about their online behavior may feel like a personal affront rather than a professional duty. There must be some form of specific teaching then to combat this irregularity ( Walji and Stanbrook, 2015). Lecturers should reiterate the guidelines in order to prevent harm to students and patients alike. Greysen *et al.* suggest that “first, do no harm” is relevant to social media use and should be at the forefront of students’ minds.

It is important for lecturers to prepare students for their professional lives; it is one of the central missions of medical education ( Ushere *et al.,* 2014). The Liaison Committee on Medical Education (LCME) in the USA point to core professional qualities such as compassion and integrity that must be developed during a student’s time at medical school ( Duke *et al.,* 2014). Social media can be used as a tool for training professionalism ( Duke *et al.,* 2014; Hennessy *et al.,* 2016). This can be achieved by creating small, faculty-facilitated groups. Duke *et al.*’s study (2014) used social media as a means of communication for the members of these groups. The students adapted quickly to this platform where they could examine, process and explore interactions that they had seen between patients and physicians. Through this they were able to increase their levels of self-reflection and preserve levels of empathy and compassion. These traits have generally been seen to decline in students entering their third year of study as well as an increase in stress and burnout ( Duke *et al.,* 2014).

There is concern over professionalism, especially from the faculty. Part 3 of the questionnaire was created to assess how students felt about this subject. This topic was one of the main barriers to social media use yet the literature does not highlight whether or not this was an issue for students themselves. Part 3 therefore is tailored to generate quantitative data on whether students know about the existing guidelines, if they have read them and if they are concerned about using social media.

### Limitations

This systematic review has a number of limitations. As social media is still a relatively new technology its growth is exponential and studies are being conducted constantly. This means that with the publication of new papers this review will soon become outdated. Additionally the decision was also made to include non peer-reviewed articles. These are more prone to bias and not as rigorous as other studies but they still added value to this review. Also due to the heterogeneous nature of the articles it was difficult to synthesise the results and implications. It was not possible to do a meta-analysis or subgroup-comparison. Although there was some quantitative data, this was limited and the majority of the information was qualitative.

As with most systematic reviews there is an element of publication bias favouring the benefits of social media rather than the negative results. At times a second author was used to minimise bias during the data extraction but this was only in the form of a review once the process had taken place. Therefore the selection bias was not completely eliminated.

## Conclusion

Social media offers a number of innovative ways to facilitate and enhance student learning. Facebook and Twitter seem to be the most popular social media platforms. Through these, lecturers can answer questions more efficiently and post relevant learning material. They can also help test students’ knowledge with multiple choice questions, pictures and diagrams. Through an understanding of Connectivism, lecturers can use social media to complement traditional learning technics and enhance their students’ education.

Social media helps bring students and staff together and can create virtual communities. As well as gaining information and valuable experience through these, they have also been found to reduce stress and anxiety all whilst maintaining levels of empathy. Increased communication also allows for faster feedback that was found in some cases to be more valuable than in class answers. Ultimately, several studies found that social media improved exam results with many students actively saying that they wanted social media to be an integral part of the medical curriculum. There is therefore a need for large scale quantitative studies so that social medias place within medical education can be verified. The questionnaire attached to this study was validated by a focus group so that it could be used worldwide to try and build quantitative evidence either in favor or against the use of social media within medical education and answer three main questions:

• What role can social media play in the medical curriculum; which platforms are most effective and for what purposes?
• How beneficial is social media to teaching; is it equal or more effective than traditional educational sources?
• What do students understand the benefits/disadvantages of academic social media platforms to be?

## Notes On Contributors

William Whyte is in his 4th year of medicine at Brighton and Sussex Medical School. In 2016 he completed a BSc (Hons) in Anatomy, Developmental & Human Biology. Throughout his studies he has had a keen interest in anatomy and medical education. This lead to his partnership with Catherine Hennessy and their subsuquent systematic review on social media use within the medical curriculum.

Catherine Hennessy completed a BSc (Hons) in Exercise and Sport Rehabilitation in 2006 after which she spent five years working privately in the sports injury field in Ireland. In 2011 Catherine moved to Edinburgh to work with the military as a rehabilitation therapist before deciding to change career paths and to university. In 2013, she graduated with a MSc in Human Anatomy which allowed her to remain at the University of Edinburgh as an Anatomy Teaching Assistant. Catherine took up a new Anatomy Teaching Fellow position at Brighton Sussex Medical School in 2015 where she is investigating how social media (and in particular Twitter) can be used to enhance the student experience in medical education.
