# Supplementary material for: Social Media use within medical education: A systematic review to develop a pilot questionnaire on how social media can be best used at BSMS
Source: MedEdPublish (2016). 2017 May 11;6:83. [Version 1] doi: 10.15694/mep.2017.000083 (PMC10885254; doi:10.15694/mep.2017.000083)
Supplement: Peer review history

# Reviewer report: Reviewer response for version 1

**DOI:** 10.21956/mep.19297.r28108

**Published:** 2017-05-20

**Authors:** Subha Ramani

**Affiliations:** 1 Harvard Medical School, Brigham and Women's Hospital

This review has been migrated. The reviewer awarded 4 stars out of 5 The authors have done a remarkable job of reviewing this literature and organizing it well for a novice social-media user (non-user) like myself. I found it easy to review the various resources, how and why they are useful and advantages and disadvantages of each. I especially found the discussion of the learning theories quite illuminating and the qualitative themes very interesting. Developing the questionnaire after review of the literature and themes is a very appropriate approach. Since current and future generations of learners utilize these resources, I need to start learning more. The paper was quite lengthy and I wish it could have been a little more concise. Otherwise, this paper would be very useful to all educators interested in using social media in their teaching and I applaud the authors for their detailed descriptions and comprehensive review.

Reviewer Expertise:

NA

No decision status is available

# Reviewer report: Reviewer response for version 1

**DOI:** 10.21956/mep.19297.r28109

**Published:** 2017-05-19

**Authors:** Veena Rodrigues

**Affiliations:** 1 Norwich Medical School

This review has been migrated. The reviewer awarded 3 stars out of 5 I think this is a valiant attempt to cover what could easily be two separate papers, the systematic review and its results and the construction, piloting and validation of a tool to inform social media use at the institution and elsewhere. One of the key strengths of this paper is the use of the PRISMA guidelines for reporting. Within the search strategy, the keywords and MeSH terms used in the Medline search are provided as is the flow diagram for study selection. However, the actual search history in Medline could have been presented fully to ensure that the methods are reproducible. The search terms included Facebook and Twitter but other social media used within medical education such as You tube, LinkedIn, etc could have been included too to ensure that the search was comprehensive. The authors extracted information from each study and summarise this in tables (appendices) to provide an overview. However, I could not find any information on the study quality assessment tool used. A validated tool such as the ‘Medical Education Research Study Quality Instrument’ (MERSQI) could have been used for assessing study quality. I think Appendix 2 could have included a score for each study quality based on quality assessment to ensure that the higher quality studies were given a higher weight in reporting the findings. The authors could also have indicated if studies used a comparator group (and what this was) in assessing benefits of social media.While minor typos are understandable but avoidable, of the six themes selected for exploration and discussion (community & interactivity, communication & feedback, learning theories, social media vs traditional didactic lectures, role of faculty and professionalism), only five were actually presented. The theme ‘social media vs traditional didactic lectures’ appears to have been missed out entirely within the discussion although it is included as a heading in appendix 2. I look forward to hearing updates from the authors on the results of their questionnaire surveys among students and how this informs curricular changes in future.

Reviewer Expertise:

NA

No decision status is available

# Reviewer report: Reviewer response for version 1

**DOI:** 10.21956/mep.19297.r28112

**Published:** 2017-05-15

**Authors:** David Taylor

**Affiliations:** 1 Gulf Medical University, Ajman, UAE

This review has been migrated. The reviewer awarded 4 stars out of 5 This is an interesting paper and I particularly liked the way the literature review informed the questionnaire design.There are three areas that I think might warrant further discussion. The first relates to the variety of uses of social media, which will each have a different impact on personal learning. In some contexts the social media are simply a way of accessing a repository of learning resources, in others they will provide personal, pastoral and arguably academic support. The second issue relates to the particular devices used, which might well frame the social media platform in use, and which has changed dramatically in the past five years or so, and is continuing to change as broadband speed and bandwidth increase. The third issue has been mentioned by other reviewers, and relates to the enthusiasm with which different groups of people adopt social media. This is an important article to read for anyone involved in understanding IT provision, and who is thinking about the next steps forward.

Reviewer Expertise:

NA

No decision status is available

# Reviewer report: Reviewer response for version 1

**DOI:** 10.21956/mep.19297.r28104

**Published:** 2017-05-13

**Authors:** P Ravi Shankar

**Affiliations:** 1 American International Medical University

This review has been migrated. The reviewer awarded 4 stars out of 5 This is a very interesting and thought provoking review. I work in an offshore Caribbean medical school and count myself as being reasonably computer savvy. The authors mention an important problem with the use of social media in medical education. The millennial generation of students is the savviest with social media and their teachers are much less savvy. The older generation is now using computers quite comfortably but still has only a passing acquaintance with social media. I do use social media for communication but am still unfamiliar with using them more widely in medical education. Social media and the internet offer relatively smaller medical schools located in small Caribbean islands access to a variety of medical education resources and can democratize medical education. With relatively good internet speeds and a student population very active on social media I am exploring how to use social media to improve student learning. Most schools while having an information technology support staff do not have an educational technologist who can help faculty become more familiar with social media and how to use it to enhance student learning. It will be a challenge to help faculty reach the level where they can help with and provide guidelines to students regarding the use of social media for education. The paper is well written and the limitations are cleanly mentioned. As Catherine Hennessy is a coauthor I did not understand the acknowledgement section where she is thanked for her contributions. I may be interested in using the questionnaire developed by the authors for a study among year 1 and year 2 students in my institution.

Reviewer Expertise:

NA

No decision status is available

# Reviewer report: Reviewer response for version 1

**DOI:** 10.21956/mep.19297.r28105

**Published:** 2017-05-12

**Authors:** Iain Keenan

**Affiliations:** 1 Newcastle University

This review has been migrated. The reviewer awarded 4 stars out of 5 This is an accomplished and interesting systematic review of social media in medical education. I have a particular interest in digital learning methods and particular social media in anatomy and medical education and I welcome practical recommendations with a strong theoretical and evidence base that support use of such technologies. The approach of utilising the review in order to develop a questionnaire is particularly effective. I look forward to reading about the results of the questionnaire in the future.A point I note in particular is with respect to the authors’ observation that there have “only” been five previous systematic reviews of social media in medical education. When considering other learning and teaching approaches in the life sciences and medical education, and noting that social media have only become popular within higher education within the last decade, 5 systematic reviews seems huge in comparison to other learning methods. This is emphasised even further by the wealth of literature (including systematic reviews and meta-analyses) with respect to social media usage beyond medical education in higher education as a whole. There are certainly further educational approaches that would benefit from this amount of attention. However, it is certainly important to identify those questions that remain to be answered with respect to social media in education, so as not to unnecessarily repeat previous work on this topic. In a similar vein, a further point I noted was the that intention is to compare social media to other learning and teaching approaches in order to identify if social media are equally effective or more effective than other methods. I would be interested to know the authors thoughts with respect to this question in terms of whether they expect that social media will be used in the future to enhance student learning through increasing the variety of available learning and teaching methods and to complement and supplement current approaches, or whether they expect social media to completely replace some existing modalities. This may influence how this question is addressed in their future work. The main approach I can currently envisage being entirely replaced by social media would be virtual learning environments, which themselves have actually been considered to be social media by some previous authors. This leads to another point with respect to the definition of social media itself. There are many definitions of social media within the literature and it is important to establish from the outset what that definition actually is. It is also beneficial not to expect that every responder or research participant will have the same definition of social media so it is important that they are either informed of the agreed definition or alternatively that their own particular definitions are identified within the research..Finally, it is always important not to fall into the “digital natives” trap when considering learning technologies including social media. Although this is an attractive idea and remains pervasive within the literature and the educational community, the notion of digital natives has been effectively discredited.

Reviewer Expertise:

NA

No decision status is available

# Reviewer report: Reviewer response for version 1

**DOI:** 10.21956/mep.19297.r28107

**Published:** 2017-05-11

**Authors:** Ronald M Harden

**Affiliations:** 1 AMEE

This review has been migrated. The reviewer awarded 4 stars out of 5 I found this a useful review of the use of social media in medical education. From the introduction alone I picked up 3 messages ..1. Of value in creating a personal learning environment ( Stephen Downes talk on PLEs at AMEE 2015), 2. Students need to learn how to make best use of SM. 3 Given the importance of role models there may be a problem with teachers serving as role models in the use of SM.I believe the future lies in a blended model and I would have liked to have seen more information about this eg how SM can contribute to a flipped classroom. However I did find of value the insights provided in the article. and recommend it as a useful read.

Reviewer Expertise:

NA

No decision status is available

# Reviewer report: Reviewer response for version 1

**DOI:** 10.21956/mep.19297.r28111

**Published:** 2017-05-11

**Authors:** Ken Masters

**Affiliations:** 1 Sultan Qaboos University

This review has been migrated. The reviewer awarded 5 stars out of 5 A very well researched and written paper. There are some minor typographical errors, and one should also note Wikipedia does have qualifying editing restrictions on some of its pages. Nevertheless, not only for the purposes of designing the questionnaire, the paper gives an excellent over view of the current use of social media in medical education. I would also like to have seen some reference to social media addiction by medical students, as this is becoming an increasing problem, but, given that the paper is already fairly expansive, it could not include everything.Finally, in the delivery of your questionnaire, just as some students were unaware that Wikipedia and YouTube were social media platforms, so many students would be unaware of Mendeley in this role, and see it only as a reference manager, so you might find yourself needing to explain its inclusion in your questionnaire.

Reviewer Expertise:

NA

No decision status is available

# Reviewer report: Reviewer response for version 1

**DOI:** 10.21956/mep.19297.r28110

**Published:** 2017-05-11

**Authors:** Barbara Jennings

**Affiliations:** 1 Norwich Medical School

This review has been migrated. The reviewer awarded 4 stars out of 5 This paper by Whyte & Hennessy describes a significant piece of scholarship about the use of social media (SM) in medical education. The systematic review (SR) results in the narrative synthesis and appraisal of data (and study design) from 27 primary studies. The paper also describes the design and revision of a questionnaire to explore student experiences and opinions of SM and explores 6 major themes; including the role of faculty, learning theories and professionalism.I enjoyed reading this paper and it addresses some of the questions posed in our editorial. I am delighted that the corresponding author, of this first paper reviewed for the themed-issue, is both a medical student and a Life Sciences graduate (see notes on contributors).The generalizable conclusions that can be drawn from the SR are limited by the quality of the primary studies (the authors appraise and describe the study designs. For example, they note in their discussion that only one of seven studies describing the use of Twitter included a control cohort). There are a lot of strengths that should be highlighted for this SR & questionnaire validation:1. Medical educationalists have embraced social media rapidly in recent years and it is important that data from any observational and interventional studies should be carefully scrutinised to inform practice. This SR includes citations that will be of great value to other academics exploring the topic, including the 5 previous systematic reviews about this subject. Whyte & Hennessy identified primary studies from the point in 2014 when the most recent previous SR manuscript was submitted. So the rationale/justification for this systematic review is clear. 2. The authors follow BEME & PRISMA guidelines in the conduct of the SR & share all data in figures and tables, either in the main body of the text or in the appendix. 3. The authors explore the limitations of the primary literature, tables 2 & 3 in the appendix are particularly useful to anyone navigating the literature. 4. The authors note the lack-of /need-for quantitative data from well-designed studies. 5. Trialling the questionnaire with a focus group allowed the authors to decide if their quantitative parameters were granular enough A few suggestions: • One principle of the SR is that clear questions should be defined as part of the protocol design; and answering a particular question is very important in the synthesis of useful information from primary studies. I think the rather open-ended objectives meant that the authors had to grapple with rather heterogeneous themes throughout. I have found that 2 things have always helped me to be very focused and clear when conducting systematic reviews (1) the use of a PRISMA checklist, http://prisma-statement.org/PRISMAStatement/Checklist.aspx from the outset, that I would publish in the appendix or submit as supplementary information with the manuscript (2) registering the protocol on the PROSPERO database; https://www.crd.york.ac.uk/PROSPERO/• Although mainly clear and interesting throughout, there were a few typos/syntax issues and the manuscript could have been a bit more succinct in a few places e.g. there is some meandering away from stated SR objectives in a few places (probably reflecting the educational interests of the authors, and frankly I still enjoyed reading about these reflections on anatomy teaching and learning). Perhaps it could have benefitted from a final edit and draft? With post-publication peer review and with copy-editing being the author’s responsibility it is always worth a careful and rigorous final edit of the manuscript. A few questions for the authors: • Do you plan to design a parallel questionnaire to explore the experiences and observations from academics too?• In the discussion you identify features of SM interventions linked to the learning of anatomy; do you think these would be generalizable to the other Life Sciences?• One of the research questions in your questionnaire is How beneficial is social media to teaching…? Given the perspectives of the participants this is designed for, I wonder if the word teaching should be replaced with the word learning.

Reviewer Expertise:

NA

No decision status is available

# Reviewer report: Reviewer response for version 1

**DOI:** 10.21956/mep.19297.r28106

**Published:** 2017-05-11

**Authors:** Trevor Gibbs

**Affiliations:** 1 AMEE

This review has been migrated. The reviewer awarded 4 stars out of 5 A very interesting and informative paper, covering a very important area- and I say that with honesty as someone who is very anti-social media!It was very healthy to read of a school researching a subject before just jumping into a methodology / technique just because it is there and assuming that all students come to medical school apparently social media savvy.I feel that this is a very good paper that should be read by all those interested in the area concerned and those considering implementing social media into their curriculum teaching and learning toolbox.If I was to improve the paper, I would suggest that in its present form it appears positively skewed towards social media and I would have liked to see more exploration on the negative aspects

Reviewer Expertise:

NA

No decision status is available
